# Re-evaluating diabetic papillopathy using optical coherence tomography and inner retinal sublayer analysis

**DOI:** 10.1038/s41433-021-01664-1

**Published:** 2021-07-09

**Authors:** Josef Huemer, Hagar Khalid, Daniel Ferraz, Livia Faes, Edward Korot, Neringa Jurkute, Konstantinos Balaskas, Catherine A. Egan, Axel Petzold, Pearse A. Keane

**Affiliations:** 1 NIHR Biomedical Research Center at Moorfields Eye Hospital NHS Foundation Trust and UCL Institute of Ophthalmology, London, UK; 2 Vienna Institute for Research in Ocular Surgery, A Karl Landsteiner Institute, Hanusch Hospital, Vienna, Austria; 3 Ophthalmology Department, Faculty of Medicine, Tanta University, Tanta, Egypt; 4 Ophthalmology Department, Federal University of Sao Paulo, Sao Paulo, Brazil; 5 Eye Clinic, Cantonal Hospital of Lucerne, Lucerne, Switzerland; 6 Dutch Expertise Centre for Neuro-ophthalmology, Departments of Neurology and Ophthalmology, Amsterdam Neuroscience, VU University Medical Center, Amsterdam, Netherlands; 7 The National Hospital for Neurology and Neurosurgery, UCLH and Queen Square Institute of Neurology, University College London, London, UK

**Keywords:** Medical research, Outcomes research

## Abstract

**Background/Objectives:**

To re-evaluate diabetic papillopathy using optical coherence tomography (OCT) for quantitative analysis of the peripapillary retinal nerve fibre layer (pRNFL), macular ganglion cell layer (mGCL) and inner nuclear layer (mINL) thickness.

**Subjects/Methods:**

In this retrospective observational case series between June 2008 and July 2019 at Moorfields Eye hospital, 24 eyes of 22 patients with diabetes and optic disc swelling with confirmed diagnosis of NAION or diabetic papillopathy by neuro-ophthalmological assessment were included for evaluation of the pRNFL, mGCL and mINL thicknesses after resolution of optic disc swelling.

**Results:**

The mean age of included patients was 56.5 (standard deviation (SD) ± 14.85) years with a mean follow-up duration of 216 days. Thinning of pRNFL (mean: 66.26, SD ± 31.80 µm) and mGCL (mean volume: 0.27 mm^3^, SD ± 0.09) were observed in either group during follow-up, the mINL volume showed no thinning with 0.39 ± 0.05 mm^3^. The mean decrease in visual acuity was 4.13 (SD ± 14.27) ETDRS letters with a strong correlation between mGCL thickness and visual acuity (rho 0.74, *p* < 0.001).

**Conclusion:**

After resolution of acute optic disc swelling, atrophy of pRNFL and mGCL became apparent in all cases of diabetic papillopathy and diabetic NAION, with preservation of mINL volumes. Analysis of OCT did not provide a clear diagnostic distinction between both entities. We suggest a diagnostic overlay with the degree of pRNFL and mGCL atrophy of prognostic relevance for poor visual acuity independent of the semantics of terminology.

## Introduction

Diabetic papillopathy is defined as uni- or bilateral optic nerve head swelling which is aetiologically thought to be directly caused by diabetes mellitus, and was first described in 1971 [1]. Initially, diabetic papillopathy was found exclusively in young patients, however, it has subsequently been shown to affect patients with diabetes in all age groups [2–5]. Whereas most descriptions were published almost 40 years ago, there is a paucity of literature on this entity in the era of modern ophthalmic imaging. Optic disc swelling in diabetic papillopathy is thought to be self-limiting without progression to optic atrophy, and without significant impact on visual function (i.e. visual field or visual acuity). The exact pathophysiology of diabetic papillopathy remains unclear, with no robust evidence for the association between diabetic retinopathy and papillopathy to date [5–8]. In bilateral cases, it has been described after alteration of glycaemic control causing rapidly changing blood glucose levels [9].

Diabetic papillopathy is a diagnosis of exclusion, hence other causes such as inflammation, infection, increased intracranial pressure, neovascularization of the disc, traction, optic disc drusen and a range of other causes of pseudo-papilloedema are primarily considered [2, 10, 11]. A neuro-ophthalmological assessment has therefore been considered an essential part of the diagnostic work-up as further testing such as neuroimaging may be warranted [12].

The similarities in the clinical presentation of diabetic papillopathy and non-arteritic ischaemic optic neuropathy (NAION) have sparked the discussion, whether diabetic papillopathy and NAION are different aetiologic and diagnostic entities or whether diabetic papillopathy is a mild form of NAION [6, 8, 13]. NAION is the most common cause of acute optic neuropathy in patients over 50 years, with diabetes mellitus as an established risk factor [14–18]. Its clinical presentation includes sudden loss of vision, swelling at the level of the optic disc, varying degree of afferent pupillary defect and dyschromatopsia as well as visual field defects [19]. A small cup-to-disc ratio, a so called crowded disc can be found in most cases [20, 21]. The swelling can be segmental or diffuse, and hyperaemic as well as pale [22]. Recurrent diabetic papillopathy is observed, and one of the risk factors for recurring NAION which only affects about 3–8% of patients [23]. However, while diabetic papillopathy and NAION have a similar structural phenotype at presentation in terms of optic disc swelling, anatomical and functional prognosis is significantly worse in NAION, eventually resulting in optic nerve head atrophy and permanently impaired visual fields or visual acuity [11]. Currently, functional assessments such as visual field testing and visual acuity assessment at presentation are used in daily ophthalmological practice to differentiate between diabetic papillopathy and NAION. Whereas visual field defects in NAION can present in various forms depending on the amount of optic nerve dysfunction, most commonly they are found nasally and inferior [24]. In diabetic papillopathy visual field defects and visual acuity are at most only mildly affected due to the absence of substantial dysfunction [11].

Optical coherence tomography (OCT) has become a staple in retinal and neuro-ophthalmological diagnostics. Quantitative assessment of peripapillary retinal nerve fibre layer (pRNFL) thickness is a modern imaging strategy to measure disease progression in various optic neuropathies like NAION or glaucoma [25, 26]. Additionally, the innermost layer of the retina, the RNFL, obtains the axons of the retinal ganglion cells, and thinning of the macular ganglion cell layer (mGCL) has been described in patients with NAION after 1–2 months, even prior to RNFL thinning [27]. The quantification of thickness of inner retinal layers may therefore help to distinguish between diabetic papillopathy and NAION.

The aim of this exploratory study was therefore to evaluate OCT changes in the pRNFL, mGCL and macular inner nuclear layer (mINL) in patients with diabetes diagnosed either with diabetic papillopathy or NAION, and thus to explore whether it is possible to differentiate between these entities in the era of modern ophthalmic imaging.

## Materials and methods

### Study setting and cohort selection

This study is a retrospective case series. An electronic medical record (EMR) search was conducted to identify all patients presented to either the Medical Retina or the Neuro-ophthalmology services at Moorfields Eye Hospital NHS Foundation Trust with concurrent diabetes mellitus and optic disc swelling between June 2008 and July 2019. We included only patients who had a Spectralis OCT scan (Heidelberg Engineering, Inc., Heidelberg, Germany) to avoid differences in measurements when comparing different OCT manufacturers and with a minimum follow-up time of 6 weeks from presentation of disc swelling. The diagnosis of either diabetic papillopathy or NAION was based on clinical examination and imaging evaluated by a neuro-ophthalmologist. Diabetic papillopathy was defined as disc swelling with no or mild optic nerve dysfunction in the presence of diabetes, the diagnosis of NAION was defined as acute or subacute visual loss in the presence of optic disc swelling with according visual field defects. Patients with optic nerve head swelling associated with other optic neuropathies than NAION or diabetic papillopathy were excluded from further analyses. Eyes with concurrent macular disease were also excluded. Spectralis OCT scans with poor imaging quality or structural changes (i.e. neovascularization of the disc, vitreous traction or peripapillary atrophy) were excluded to ensure accurate segmentation and quantification of the pRNFL thickness. Approval for data collection and analysis was obtained from the Institutional Review Board at Moorfields (ROAD17/031). The study adhered to the tenets set forth in the Declaration of Helsinki. The updated Apostel 2.0 OCT reporting guidelines were followed and recommended terminology used [28]. All OCT scans were quality controlled using the OSCAR-IB criteria [29].

We extracted demographic and clinical information such as best-corrected visual acuity (BCVA) at the time of presentation and final visit concurrent to the OCT from the EMR. In addition, ophthalmic imaging material (i.e. colour fundus photography, fluorescein angiography (FA) and OCT) was reviewed.

### Image grading

OCT scans were obtained using a spectral-domain OCT device (Heidelberg Engineering, Germany). Thickness measurements of the pRNFL, mGCL and mINL were extracted as quantified on OCT scans. The pRNFL was measured using a circle scan with 3.45 mm diameter centred on the optic disc. The pRNFL was automatically segmented (software version 6.7.13.0), and a “six sector” map was generated providing global, temporal, temporal-superior, temporal-inferior, nasal, nasal-superior and nasal-inferior sector measurements of the pRNFL thickness. The SD-OCT age-adjusted reference database was used to calculate deviations from normal means of the measurements. Thickness measurements of pRNFL are indicated categorically in green, representing “within normal limits” (within 95% normal distribution), yellow representing “borderline” (between the lower 95.0% and the lower 99% of normal distribution) or red representing “outside normal limits” (below the lower 99% of normal distribution), respectively [19].

Volume measurements of the macular mGCL and the mINL were acquired using a 25 × 30-degree macular volume scan with 25 or 49 B-scans centred on the fovea. The layers of the retina were automatically segmented by the Heidelberg HRA/Spectralis software. Thickness and volume measurements of the mGCL and mINL were evaluated within the EDTRS grid-overlay including three circles centred at the fovea ((i) 1 mm, (ii) 2.22 mm, (iii) 3.45 mm in diameter respectively). The assessment included global mean thickness measurements in (i), sector-specific thickness measurement within four separate quadrants in (ii) and (iii), and a global volume measurement of all quadrants combined. In case of segmentation error, the segmentation was manually corrected. Measurements of the pRNFL were compared to published normative data while mGCL and mINL measurements were compared to a control group of normal subjects [30]. Macula normative data was generated from SD-OCT images of 34 healthy eyes of 17 healthy volunteers or patients with no known retinal disease, BCVA of 20/20 and normal intraocular pressure [30].

Two masked retinal specialists (HK, JH) independently reviewed colour fundus photography, FA and OCT images for evaluation of optic disc swelling at presentation and changes at last visit. Disagreements between graders were adjudicated by a senior retina specialist (PAK).

### Statistical analysis

Statistical analysis was done using R studio (version 1.2.1335) for R [31]. Snellen visual acuity was converted to ETDRS letters [32]. Descriptive statistics included mean, SD and percentages. Pearson´s correlation coefficients were calculated for the association between mGCL, mINL and visual acuity.

## Results

**Fig. 1:**
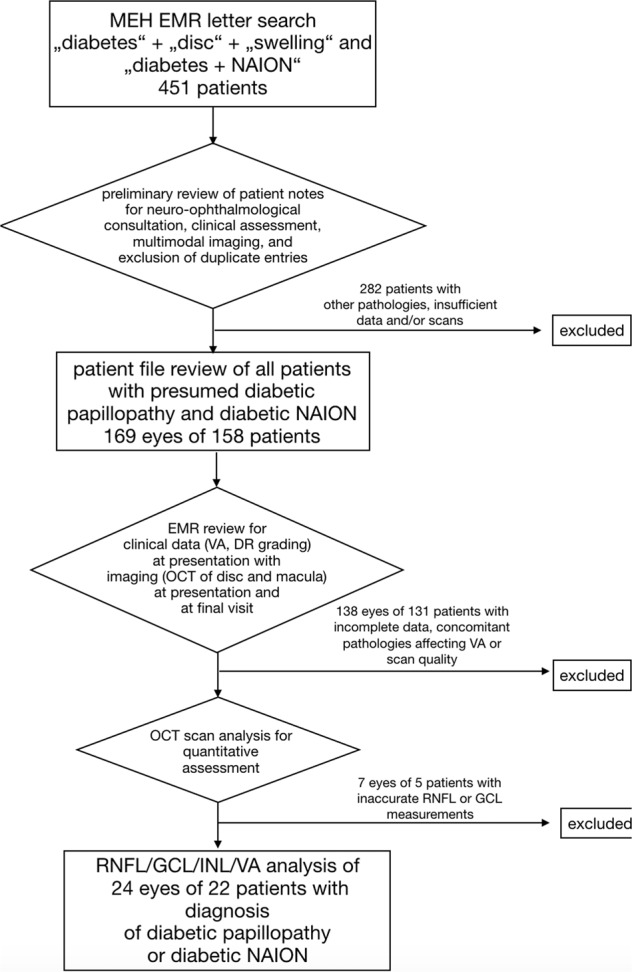
Patient selection. Flow diagram: Data labelling of the Moorfields EMR dataset.

Between June 2008 and July 2019, 451 patients with diabetes presented to the Moorfields Eye Hospital NHS Foundation Trust with optic disc swelling. In 169 eyes of 158 patients, diabetic papillopathy or NAION was diagnosed by a comprehensive neuro-ophthalmological assessment including visual fields and OCT, or, if indicated, magnetic resonance imaging after exclusion of other causes of optic disc swelling. Of these, 37 eyes of 32 patients underwent Spectralis OCT imaging during their follow-up. We excluded six eyes of five patients with macular diseases such as macular oedema, or photoreceptor atrophy. Furthermore, we excluded seven eyes of five patients in whom there was an algorithm failure OCT of retinal layer segmentation which could not be corrected manually, i.e. due to large neovascularization of the disc, traction at the disc, peripapillary atrophy or decentred scans [29]. As a result, a total of 24 eyes of 22 patients passed consensus quality control and were included in our study. (Fig. 1).

### Patient demographics and clinical information

Of 22 included patients, 11 were female (50%). Patient’s age at presentation ranged from 26 to 81 years, with a mean age of 56.5 years (SD ± 14.85 years). 17 patients (70.83 %) had no diabetic retinopathy, five patients (20.83%) had mild non-proliferative diabetic retinopathy (R1), one patient (4.17%) had moderate to severe diabetic retinopathy (R2), and one patient (4.17%) had stable proliferative diabetic retinopathy (R3S). The mean follow-up period was 215.77 (SD ± 161.79 days, range 89–818). Macula normative data was generated from SD-OCT images of 34 eyes from 17 individuals with no known optic disc or macula pathology and BCVA of 6/6 (0.00 logMar), who underwent imaging at Moorfield Eye Hospital. Mean age was 37.88 years (SD ± 16.49 years, range 17–72) (Table 1) and the biggest proportion (82%) of normal control were females.

**Table 1:** Patient’s demographics and clinical information.

Female, *n* (%)	11 (50%)
Age, mean (±SD), range, in years	56.5 (±14.85), 26–81
DR^a^ grading, *n* (%)	
no DR (R0)	17 (70.8%)
mild DR (R1)	5 (20.8%)
moderate DR (R2)	1 (4.2%)
stable treated DR (R3S)	1 (4.2%)
Mean follow-up duration (±SD) in days	216 (±162)
Mean BCVA^b^ at baseline (±SD) in ETDRS letters	64.78 (±20.59)
Mean BCVA^b^ at the last follow-up visit (±SD) in ETDRS letters	60.65 (±25.28)
Mean BCVA^b^ decrease from baseline to follow-up visit (±SD) in ETDRS^c^ letters	−4.13 (±14.27)
Mean global pRNFL^d^ measurements at the last follow-up visit (±SD) in µm	66.26 ± 31.80
Mean mGCL^e^ volume at the last follow-up visit (±SD) in mm^3^	0.27 ± 0.09
Mean mINL^f^ volume at the last follow-up visit (±SD) in mm^3^	0.39 ± 0.05

^a^*DR* diabetic retinopathy.

^b^*BCVA* best-corrected visual acuity.

^c^*ETDRS* Early treatment diabetic retinopathy study.

^d^*pRNFL* peripapillary retinal nerve fibre layer.

^e^*mGCL* macular ganglion cell layer.

^f^*mINL* macular inner nuclear layer.

### OCT changes of the pRNFL during follow-up

**Fig. 2:**
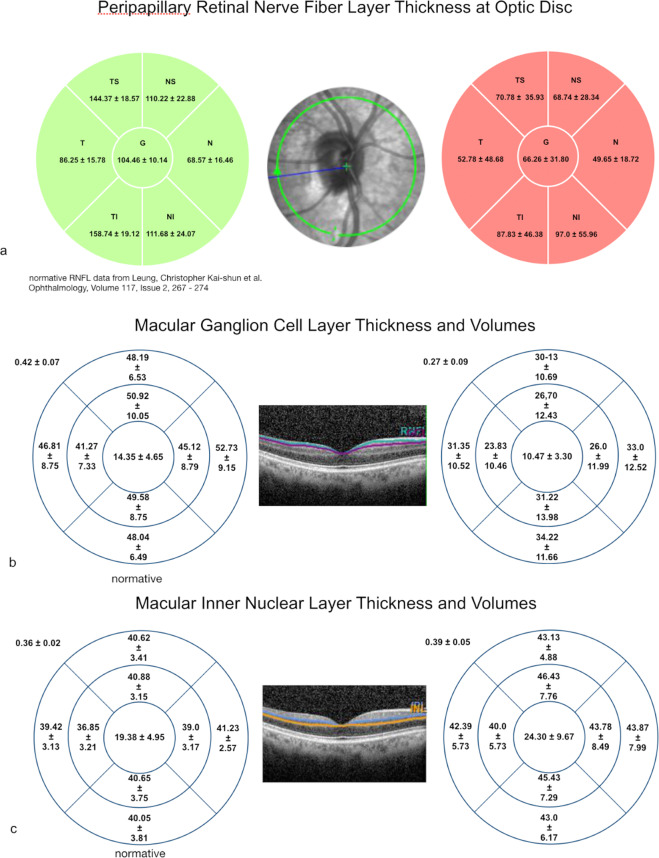
Sublayer analysis. **a** Comparison of normative control peripapillary retinal nerve fibre layer (pRNFL) data (above left) and mean pRNFL thickness (±SD) of the optic disc in patients with non-arteritic anterior ischaemic optic neuropathy (NAION) and diabetic papillopathy (above right). A decrease of mean pRNFL is visualised by colour coding (red signifies pRNFL atrophy). **b** Comparison of mean macular ganglion cell layer (mGCL) thickness and volumes (±SD) of normative control data set (Moorfields Eye Hospital, London) and patients with diabetic papillopathy and NAION. The mean macular inner nuclear layer (mINL) thickness and volumes (**c**) of our cohort and the normative group showing that it is not affected.

Qualitative pRNFL thinning was noticed in all patients at the final visit. It was ranging from at least one segment involved to all the segments involved and from borderline to significant thinning. The mean global pRNFL thickness was 65.88 ± 31.16 µm compared to 104.46 ± 10.14 µm of global pRNFL of the normative data. The mean pRNFL measurements of the global, temporal-superior, nasal-superior, temporal, nasal, temporal-inferior and nasal-inferior sectors were summarised in comparison to the normative data in Fig. 2.

### OCT changes of the mGCL and mINL during follow-up

Diffuse or sectoral mGCL thinning was observed in all cases at the final visit, whereas no changes could be detected in the mINL qualitatively. The mean thickness in the centre 1 mm, four quadrants within the inner and outer rings of the grid overlay as well as global volume measurements were summarised in Fig. 2 and compared to the normative control group. The mean global volume of the mGCL was 0.27 ± 0.09 mm^3^ compared to 0.42 ± 0.07 mm^3^ in the control group, while the mean global volume of the mINL was 0.39 ± 0.05 mm^3^ compared to 0.36 ± 0.02 mm^3^ in the control group.

### Visual acuity changes

The mean baseline BCVA of our cohort was 64.78 ± 20.59 letters. Of those four patients had BCVA ≥ 85 (Snellen 6/6). The mean BCVA at last visit was 60.65 ± 25.28, five of them had BCVA ≥ 85 (Snellen 6/6). The mean changes in BCVA during follow up was −4.13 ± 14.27 letters. The proportion with eyes with preserved BCVA was 37.5% (9 eyes), deterioration was noted in 37.5% (9 eyes), while improved BCVA during follow up was noticed in 25% (6 eyes). The BCVA at the follow-up visit was significantly correlated with the mean global volume of the mGCL (rho 0.74, *p* < 0.001) Supplementary Fig. 1). No significant correlation was found with neither the mean global volume of the mINL nor the mean global pRNFL thickness (rho 0.07, *p* 0.75), (rho 0.21 *p* 0.33) respectively.

### Case study

#### Case 1

**Fig. 3:**
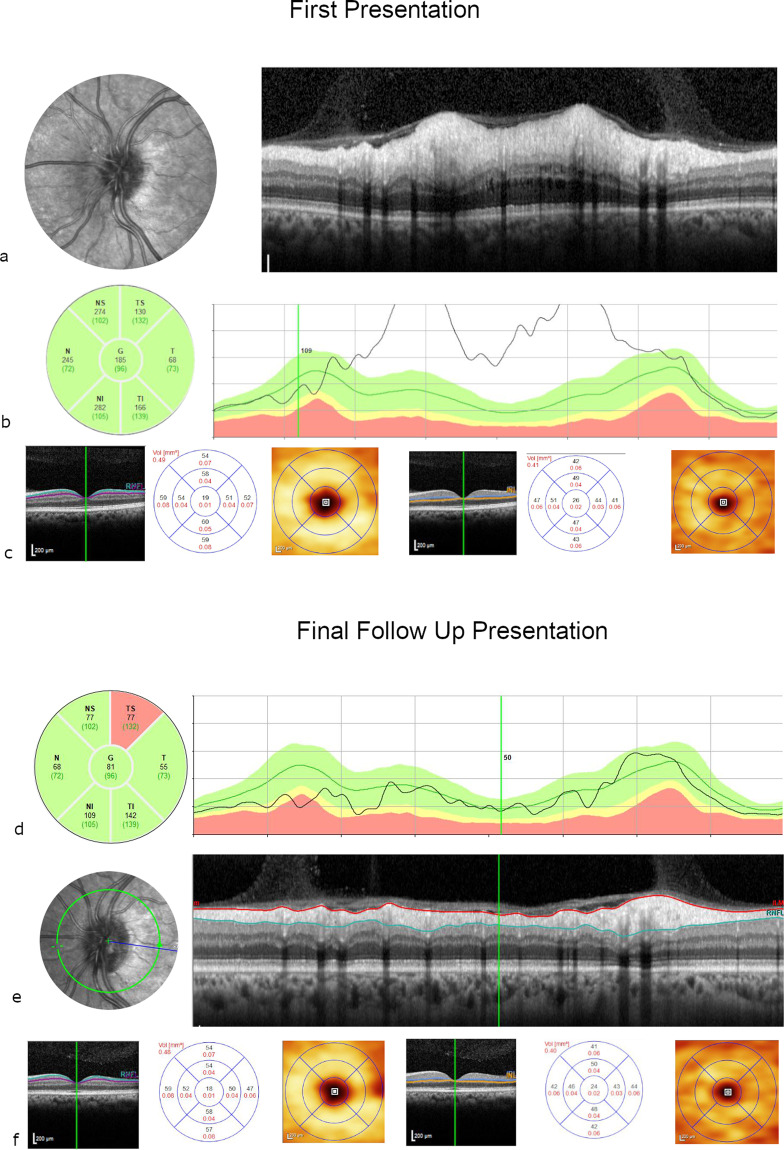
Case 1. Confocal scanning laser ophthalmoscopy (near infrared, top left) and optical coherence tomography (OCT) (top right) images of the peripapillary retinal nerve fibre layer (pRNFL) at presentation (**a**) and after 8 months (**c**) and macular ganglion cell layer/macular inner nuclear layer (mGCL/mINL) volumes and thicknesses at presentation (**b**) and after 8 months (**d**). A 55-year old patient with diabetes (type 2) with diabetic papillopathy in the left eye, presented with good visual acuity of 20/25 (80 ETDRS letters) and segmental optic disc swelling (**a**) on OCT and near infrared image. The visual acuity remains excellent after the disc swelling has resolved (20/20 snellen, 85 ETDRS letters). After 8 months, a small ischaemic defect of the pRNFL in the superotemporal optic disc segment can be demonstrated (**c**), that also leads to a wedge defect in the mGCL at the macular scan (**d**) compared to measurements at presentation (**b**). The mINL thickness remains preserved over time (**b**, **d**).

A 55-year old patient with poorly controlled type 2 diabetes and bilateral mild non-proliferative diabetic retinopathy was referred from the United Kingdom Diabetic Retinopathy Screening Service after identification of left optic disc swelling. This was confirmed by a circular OCT scan of the optic disc demonstrating significant thickening of the pRNFL. At presentation, the visual acuity in the left eye was 80 ETDRS letters (Snellen 20/25) with preserved colour vision. The visual fields on confrontation were full with normal pupillary reactions. On fundus examination, the optic disc appeared hyperaemic with radial telangiectatic vessels. Humphrey visual field test revealed subtle changes without evidence of an altitudinal field defect. The diagnosis of diabetic papillopathy was made after exclusion of other causes of optic neuropathy based on blood tests, imaging and neuro-ophthalmological consultation. Eight months later, optic disc swelling had resolved with restoration of normal visual acuity (20/20 Snellen, 85 ETDRS letters). However, significant sectoral thinning of pRNFL was detected superotemporally. Additionally, there was a notable sectoral defect in the mGCL on the macular OCT scan while the mINL remained preserved consistent with previous observations [33]. (Fig. 3).

#### Case 2

**Fig. 4:**
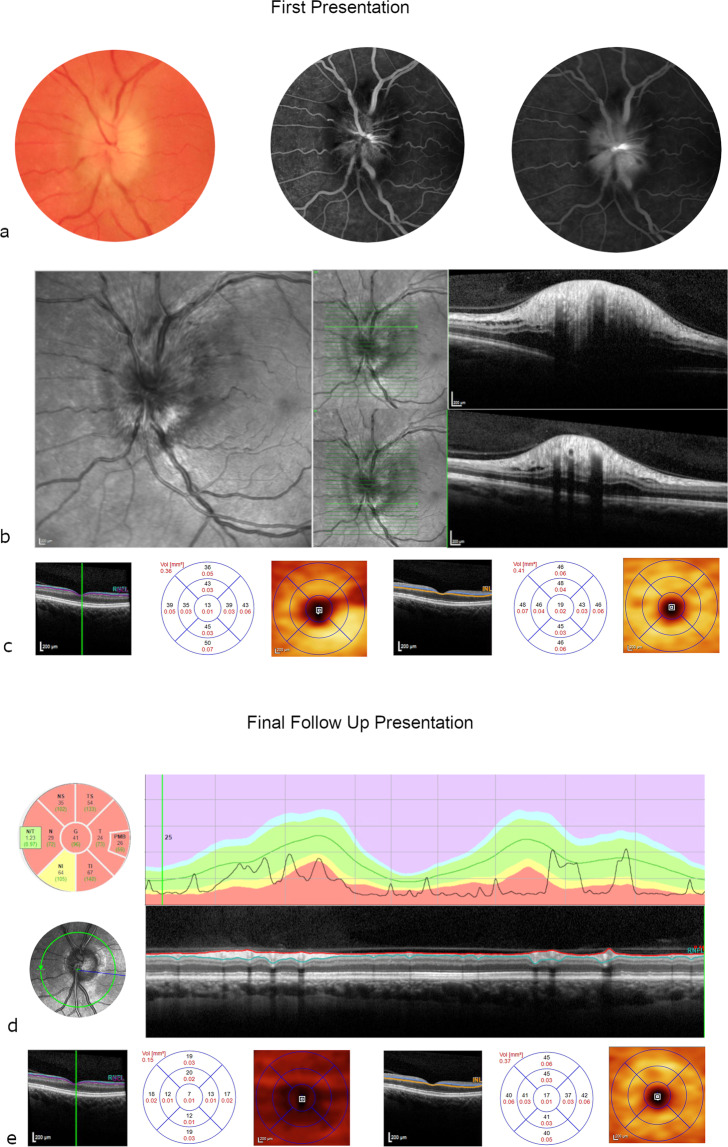
Case 2. Colour photo/fluorescein angiography (FA) (early/late phase) (**a**), optical coherence tomography (OCT)/near infrared at presentation (**b**), macular ganglion cell layer/macular inner nuclear layer (mGCL/mINL) at presentation (**c**), peripapillary retinal nerve fibre layer (pRNFL) and OCT disc at follow-up (**d**), mGCL/mINL at follow-up. Fifty-one year old female with type 2 diabetes and NAION. At presentation the visual acuity was 75 ETDRS letters (Snellen 20/32), with diffuse optic disc swelling seen in the colour photo, infrared and OCT scan of the optic disc and diffuse late hyperfluoresence in FA. After 8 months follow-up the visual acuity dropped to 20/200 and there is evident disc atrophy in the pRNFL thickness map. mGCL thinning can be observed, whereas the mINL volume is preserved.

A 51-year old female with hypertension and type 2 diabetes presented to Moorfields Emergency Department with sudden painless left visual deterioration. On examination, the visual acuity was 75 ETDRS letters (Snellen 20/32) in the left eye. A swollen optic disc was clinically evident, confirmed by OCT scanning, and by FA, which showed late diffuse leakage. Visual field testing demonstrated an inferior altitudinal field defect. The diagnosis of NAION was confirmed by a specialist neuro-ophthalmologist based on the clinical evaluation and a normal MRI scan. After 8 months follow-up, the visual acuity dropped to Snellen 20/200, with evident disc pallor and significant diffuse thinning of the pRNFL. Diffuse mGCL thinning could be observed. Consistent with what is observed in NAION the mINL was not affected in the macular thickness map [33]. A diagnosis of recurrent NAION was made. (Fig. 4).

#### Case 3

**Fig. 5:**
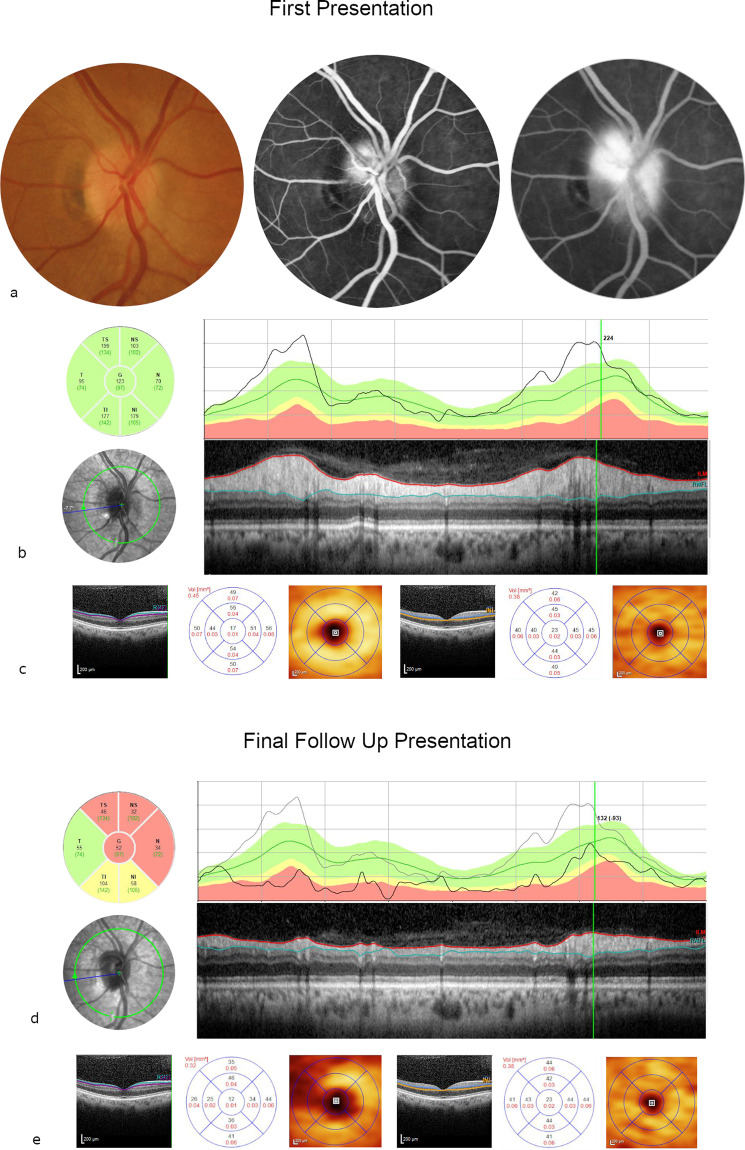
Case 3. Colour photo/fluorescein angiography (FA) (early/late) (**a**), optical coherence tomography (OCT)/peripapillary retinal nerve fibre layer (pRNFL) (**b**), macular ganglion cell layer/macular inner nuclear layer (mGCL/mINL) (**c**) at presentation, OCT/pRNFL (**d**) and mGCL/mINL (**e**) at follow-up. A 46 year old female patient with type 2 diabetes presented with presumed diabetic papillopathy because of good initial visual acuity of 20/20 (85 ETDRS letters) and disc swelling (blurred disc margins in the colour photography). The FA showed late evident sectoral staining. After 4 months the disc swelling has resolved with unchanged visual acuity of 20/20 (85 ETDRS letters). pRNFL thinning affecting the entire optic disc except the temporal segment and mGCL loss at the macula demonstrate ischaemic damage, while the volume of the mINL was unchanged.

A 46-year old female patient with poorly controlled type 2 diabetes developed sudden blurring in the lower half vision in her right eye. On examination, the right visual acuity was excellent (Snellen 20/20, 85 ETDRS letters), with unaffected colour vision. Pupil examination revealed a right relative afferent pupillary defect and Humphrey visual field testing showed a lower hemifield defect. Sectoral optic disc swelling was observed, without signs of diabetic retinopathy on fundus examination. There was evidence of late sectoral leakage on FA. The diagnosis of diabetic papillopathy was presumed because of good initial visual acuity and after exclusion of other causes of optic disc swelling at the neuro-ophthalmological consultation. After 4 months of observation, the disc swelling had resolved with preserved visual acuity of 85 ETDRS letters (Snellen 20/20). However, diffuse pRNFL thinning affecting the entire optic disc with the exception of the temporal segment was evident on OCT. There was also mGCL loss at the macula, again without changes in the mINL as described [33]. (Fig. 5).

## Discussion

Diabetic papillopathy is a term historically used for patients with no or only mild optic nerve dysfunction using good visual acuity as a marker for diagnosis [2]. Whereas the hypothesis that diabetic papillopathy is a milder form of NAION has been discussed for over 35 years, our findings provide further insight into the ischaemic nature of diabetic papillopathy and NAION. In our study, using OCT segmentation analysis of patients with both diabetic NAION and presumed diabetic papillopathy, similar patterns of pRNFL and mGCL thinning have been noted in all cases regardless of visual acuity. Furthermore, mGCL thickness is the determining factor on the visual acuity with a strong positive correlation observed. Thus, we propose that diabetic papillopathy is a potentially mislabelled benign entity as ischaemic damage sequences can, as our data suggest, always be found after disc swelling with OCT.

In our study, we were able to show that after the initial optic head swelling has settled, both diseases share the same structural features. In a study by Kupersmith et al., early mGCL thinning has been shown to precede the pRNFL thinning in patients with NAION [34]. In all of our cases of both NAION and presumed diabetic papillopathy, pRNFL and mGCL thinning was observed. This supports the hypothesis that diabetic papillopathy is indeed an ischaemic neuropathy with objectively quantifiable thinning of the inner retinal layers and therefore not benign.

Interestingly, the mINL remained unaffected in all of our patients. This is such a consistent clinical observation that it was proposed to include this to “three red lines” which aid with pattern recognition for the differential diagnosis of OCT [33]. There are two anatomical reasons for this observation. First, the dense network of bipolar, amacrine and horizontal cells with an extensive synaptic tree in the INL, providing more neuronal network integrity and plasticity compared to the other neurons in the otherwise “hard-wired” optic pathway and functioning as a physiological barrier against trans-synaptic retrograde degeneration in optic neuropathies [35]. Second, there are important differences in the vascularisation between the optic disc and peri-macular area. Blood supply from the inner retina, through the superficial vascular plexus (SVP) is high at the optic disc, but decreased towards the macula, to be completely absent from the “foveal avascular zone” which is supplied by the choroidal plexus and deep vascular plexus (DVP). The SVP and DVP are located at the superior and inferior border of the INL and connected by capillaries. It is plausible that these capillaries provide sufficient blood supply to the INL to prevent ischaemic damage with a vascular occlusion only affecting capillaries supplying the optic disc as described in NAION.

The leading clinical feature of diabetic papillopathy is thought to be the absence of optic nerve dysfunction, and therefore preserved visual acuity [2]. In NAION, the visual acuity may vary from 20/20 Snellen to poor visual acuity [36]. We could observe excellent final BCVA with 20/20 or more in five patients, despite the pRNFL and mGCL thinning being noted in all our patients. This suggests that good visual acuity is not the sole indicator of the visual function for those patients. In a retrospective study analysing 386 eyes, Hayreh et al. showed that 49% of their patients had a visual acuity of 20/30 or better within the first 2 weeks of presentation [36]. In our cohort, the proportion of patients who maintained 20/30 Snellen or better was 37.5 %. This difference, 49% vs 37.5%, could be explained by the longer duration of follow-up in our study. Considering the mean change in the visual acuity, more than half of our patients (62.5%) have either preserved or improved BCVA at last visit while in only 37.5%, deterioration of visual acuity was observed.

We observed asymptomatic disc swelling in almost 17% of our cohort (in four referred patients for optic disc swelling, while the BCVA was 20/20 or more). It has been described earlier in a study by Almog and Goldstein when observing patients who converted to NAION defined as subjective visual loss, visual field defect with or without decreased VA or the presence of an afferent pupillary defect [13]. Furthermore, asymptomatic disc swelling has also been described by Hayreh et al. as incipient NAION, which was defined as disc oedema without subjective or objective visual loss being observed in eyes with a history of NAION in the fellow eye [37]. Of interest, 83% and 63% of patients in these studies respectively had diabetes, and would fit into the characteristics of the commonly described diabetic papillopathy. As all of these studies were conducted prior to the use of OCT, it is unsure whether the optic disc dysfunction was partial and which optic disc segments were affected ultimately. Since the introduction of OCT, vitreous traction at the optic disc can be added to the list of causes for asymptomatic disc swelling. And presence of optic disc drusen and peripapillary hyper-reflective ovoid mass like structures have been added to the list of risk factors for NAION, particularly in the young patient [38].

To illustrate the influence of the structural changes on the visual outcomes, OCT segmentation analysis in correlation to the final BCVA was evaluated. The mean pRNFL thickness measurements at the final visit, despite evident thinning in all patients when compared to normative data, did not show any correlation with the visual acuity findings. This can be explained by sectoral thinning sparing the papillomacular bundle which is key to foveal vision. This line of argumentation is consistent with the clinical observation of preserved colour vision in NAION. Even if NAION leads to a central visual field defect, patients are typically still able to correctly detect colours in the remaining sectors. Of interest, in our cohort, the visual acuity was strongly positively correlated to the mean global macular mGCL thickness (rho 0.74, *p* < 0.001). As expected, when comparing the mINL thickness to the visual acuity, no correlation could be found. Our results suggest that visual outcome in diabetic papillopathy and NAION is influenced by mGCL thickness, with thinner mGCL associated with worse visual outcomes. While the RNFL not only contains the axons of the GCL but also significant proportions of glial and vascular components, this could explain why the mGCL thinning measured by spectral-domain OCT would serve as a more precise modality when assessing optic nerve function [39].

Both diabetic papillopathy and NAION share common characteristics in their clinical presentation, and differentiation between both based on the initial visual acuity or the final visual outcome is not definite, as suggested by OCT segmental analysis. Preservation of the INL is one of these features [33]. The disc swelling in NAION was reported to be either sectoral in 25% or diffuse in 75% [19], which leads to a loss of the nerve fibres at the disc in the area of the swelling. The amount and location of disc swelling consequently is accountable for the location of the visual field defect and reduction in visual acuity. In case of a generalised disc atrophy, a loss of visual acuity can be severe. In contrast to this, if, however, the temporal part of the optic disc is intact, the visual acuity testing might not show any loss as the papillomacular bundle is preserved, even though wide ischaemic changes can be found elsewhere.

Diabetic papillopathy is generally referred to as a mild condition in terms of optic nerve dysfunction. We, however, observed that if in cases with mild sectoral swelling and excellent visual acuity, pRNFL changes following the same patterns as in NAION. As the term diabetic papillopathy is mainly historically, and a clear distinction to NAION can not be observed when applying OCT scans, no clear argument can be made to keep using a potentially confusing description. Patients with ischaemic optic nerve disease need the best possible management of their systemic risk factors, which is usually undertaken by their treating physicians. For example, phosphodiesterase type five inhibitors like sildenafil used to treat erectile dysfunction, a common condition in patients with diabetes, are contraindicated in patients with NAION. Patients with other common optic neuropathies like glaucoma, where no signs of visual field defects are visible, are labelled as having pre perimetric glaucoma. One might argue that there is no such thing as “benign optic neuropathy”. This should be remembered for patient management.

Limitations of our study are mainly due to the retrospective nature of this exploratory small case series. By choosing only a single device for OCT analysis, we have a limited number of included patients, this, however, makes the OCT findings more reliable. The small number of patients included is attributed to not all patients having macular and disc OCT scans during the follow-up, as this is not considered part of the clinical standard. As there are no definite diagnostic criteria for diabetic papillopathy, we were dependent on the clinical judgement of the treating ophthalmologists and the documented visual acuity at presentation. Patients were reviewed by several neuro-ophthalmologists. The integrated care pathway for these patients includes assessment by the stroke service and patients underwent neuroimaging. The control group for the macular scans is not age-matched or laterality matched. The cup-disc ratio was not included in the analysis.

The omnipresence of OCT-based quantification of retinal morphology allows to objectively evaluate optic nerve head diseases in higher granularity. Both entities, diabetic papillopathy and NAION, showed thickening of the peripapillary RNFL with macular preserved GCL and macular INL at presentation. This was followed by atrophy of the pRNFL and mGL, but preservation of the INL. Regardless of the amount and location of optic disc swelling, the grade of diabetic retinopathy and the initial visual function, both entities have variable outcomes. Thus strikingly, we could not observe any distinguishing structural imaging biomarkers for NAION and diabetic papillopathy either acutely or later in the disease course. Considering the practical implications for patient management, our practical advice is to manage both conditions equally which includes rigorous management of cardiovascular risk factors.

### Summary

#### What was known before

- Diabetic papillopathy and non-arteritic anterior ischemic optic neuropathy (NAION), although described as distinct entities, can present with identical clinical findings in the initial presentation, and overlaps have been described.

#### What this study adds

- Optical coherence tomography (OCT) analysis of inner retinal layers show loss of the peripapillary retinal nerve fibre layer (pRNFL) and macular ganglion cell layer (mGCL) in all cases of diabetic papillopathy and NAION regardless of visual acuity after resolution of optic nerve swelling; however, no thinning of the macular inner nuclear layer (mINL) can be observed. OCT-based quantification of retinal morphology allows optic nerve head diseases evaluation in higher granularity, showing no distinguishing biomarkers for NAION and diabetic papillopathy.

## Supplementary information

Supplementary Figure 1
