# Supplementary figures and images for: Re-evaluating diabetic papillopathy using optical coherence tomography and inner retinal sublayer analysis
Source: Eye (Lond). 2021 Jul 9;36(7):1476–85. doi: 10.1038/s41433-021-01664-1 (PMC9232525; doi:10.1038/s41433-021-01664-1)

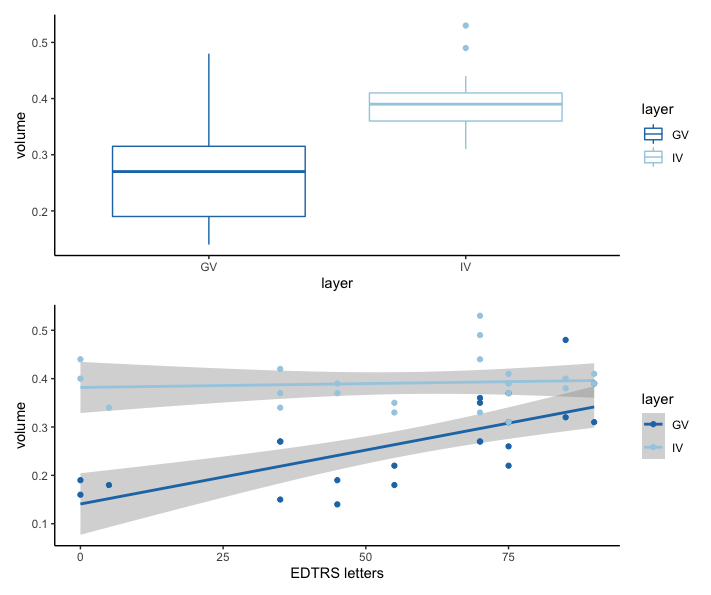

Supplement: Supplementary file 1 — Supplementary Figure 1 [file 41433_2021_1664_MOESM1_ESM.png]
